# A long history of dense deposit disease

**DOI:** 10.1186/s12886-018-0853-8

**Published:** 2018-09-14

**Authors:** Alan Cunningham, Ajay Kotagiri

**Affiliations:** Sunderland Eye Infirmary, Queen Alexandra Road, Sunderland, UK

**Keywords:** Dense deposit disease, Mesangiocapillary glomerulonephritis, MPGN, Retinal drusen, Kidney failure

## Abstract

**Background:**

Dense Deposit Disease is a rare condition affecting the Bruch’s membrane and the glomerular basement membrane. We report the progression of the ocular manifestations over a 30 year follow up period, longer than any previous report.

**Case presentation:**

A 44 year old male presented with pigmentary changes at the macula noted by his optician. Best corrected visual acuity at presentation was good in both eyes. Fundoscopy showed pigmentary changes and drusen, and investigation using intravenous fundus fluorescein angiography did not demonstrate any choroidal neovascular membrane. The patient subsequently developed renal failure and received a dual renal transplant. The transplanted kidneys also failed over the coming year. The patient’s vision gradually deteriorated and comparison between the images in 2010 and 1985 demonstrated a clear progression of the macula changes. Optical coherence tomography showed multiple subretinal hyper reflective drusenoid deposits. These deposits were also noted to be autofluorescent on blue auto-fluorescence. The young age at presentation of drusen, combined with the history of recurrent kidney failure and progression of subretinal deposits led to a diagnosis of dense deposit disease.

**Conclusions:**

Dense deposit disease is a rare condition affecting Bruch’s membrane, but should be considered in the differential diagnosis of any patient under the age of 50 years presenting with drusen.

## Background

Retinal drusen are a common finding in older patients and are usually attributed to Age Related Macular Degeneration. However, in patients under 50 years of age it is particularly important to consider other aetiologies. Dense deposit disease is a rare condition that causes drusenoid deposits within Bruch’s membrane and the glomerular basement membrane and leads to end stage renal failure in 50% of patients [1].

The current literature includes a cohort of four patients followed up at 10 years, and a case with a renal history of 48 years, but only reviewed in the ophthalmological services for 6 years. The case presented here documents the progression of the clinical findings and visual acuity over a 30 year period.

## Case presentation

**Fig. 1:**
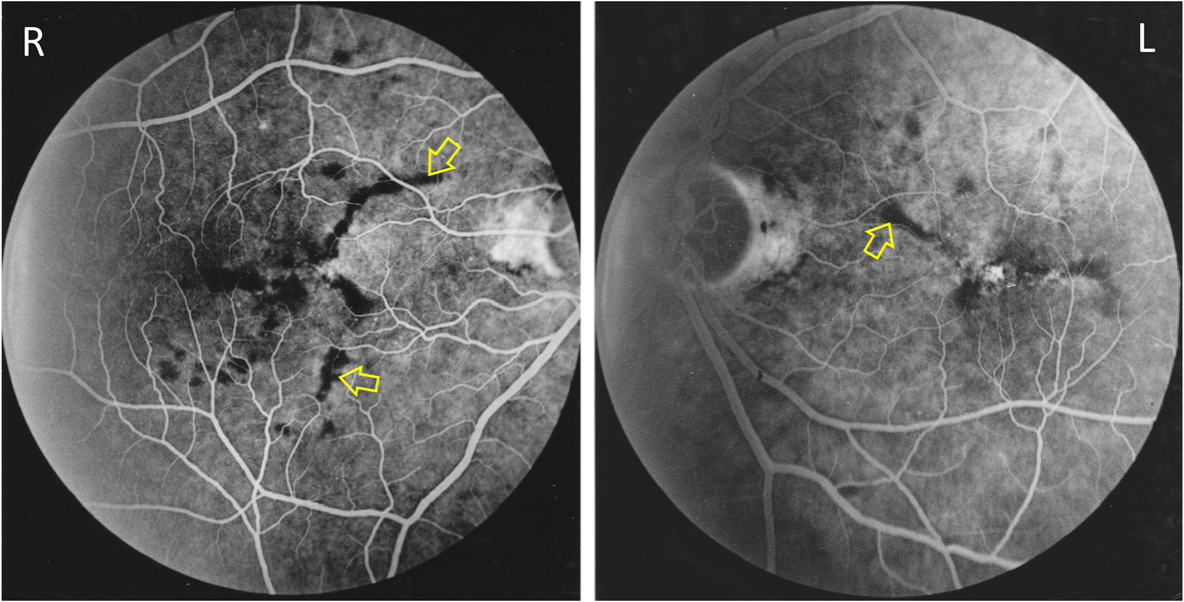
IVFA at presentation (1985) demonstrating bilateral drusen seen as a blocking defect (yellow arrows) which is worse in the right eye

A 75 year old Caucasian male with a long history of retinal changes was seen in the clinic. He had initially presented 31 years earlier, in 1985, with pigmentary changes at the macula noted by his optician. At the time his best corrected visual acuity (BCVA) was 6/9 in the right eye and 6/6 in the left eye. Fundoscopy showed pigmentary changes and drusen which were more easily visible on intravenous fundus fluorescein angiography (IVFA) (Fig. 1). There was no evidence of any choroidal neovascular membrane (CNVM) on any of the images. The patient underwent multiple further IVFA examinations, each time demonstrating no active leak. His retinal appearance was monitored, with no conclusive diagnosis made, nor treatment available.

**Fig. 2:**
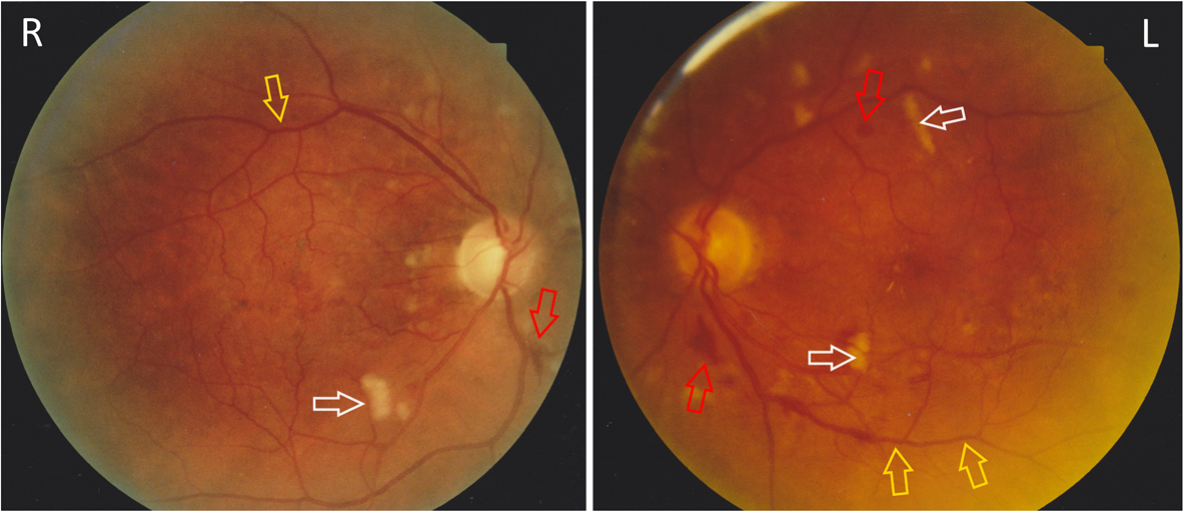
Bilateral fundus colour photographs demonstrating cotton wool spots (white arrows), venous beading (yellow arrows) and haemorrhaging (red arrows), consistent with hypertensive retinopathy

Ten years later ongoing review identified that the patient had raised intraocular pressure along with optic disc changes and a diagnosis of glaucoma was made with appropriate treatment initiated. Subsequently, in 2001, the patient was diagnosed with hypertension (186/110 mmHg) which, at the time, was thought to be essential hypertension. Retinal examination demonstrated cotton wool spots and haemorrhages, consistent with hypertensive retinopathy (Fig. 2). He was also noted to have elevated serum urea and creatinine levels which were assumed to be related to the diagnosis of hypertension.

Following bilateral cataract surgery in 2003, which was complicated by posterior capsule rupture in the left eye, the patient was only able to achieve a BCVA of 6/36 in each eye. Due to inadequate control of IOP left sided trabeculectomy was performed in 2005.

At the same time, aged 64 years, the patient’s urea and creatinine levels were recorded as 20.5 mmol/L (normal 2.8–7.2 mmol/L) and 474umol/L (normal 60-105umol/L) respectively and he started regular haemo-dialysis for end stage renal failure shortly afterwards. He then underwent dual renal transplant in 2009 and was initiated on systemic immunosuppression (Tacrolimus 3 mg/day) which he continues to this day. The biopsy results from the explanted kidneys are unfortunately not available. Subsequently both transplanted kidneys failed over the coming year, with no identified cause, requiring the patient to undergo bilateral nephrostomies.

**Fig. 3:**
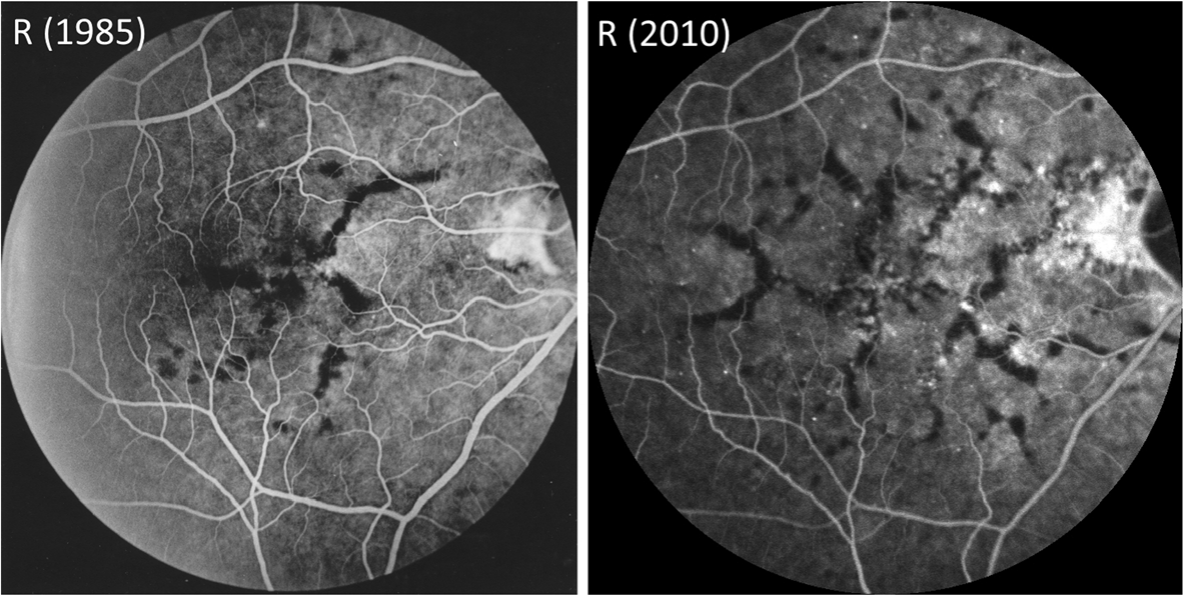
Comparison of the IVFA of the right eye at presentation in 1985 and at re-referral in 2010

In June 2010 the patient was referred back to the Ophthalmology services, aged 69, with a presenting vision of 52 ETDRS (Early Treatment Diabetic Retinopathy Study) letters in the right eye and 35 letters in the left eye. He was again noted to have bilateral changes at the maculae which were, at the time, attributed to possible age related macular degeneration, and a small area of possible sub-retinal fluid. Comparison was made between the IVFA in 1985 and the IVFA at re-referral showing a significant increase in the number and distribution of the drusen, but no vascular leakage (Fig. 3). The poor vision in the left eye was found to be secondary to advanced glaucoma and significant changes at the macula. No treatment was appropriate and the patient was monitored for 5 years with repeated Optical Coherence Tomography (OCT) scans.

**Fig. 4:**
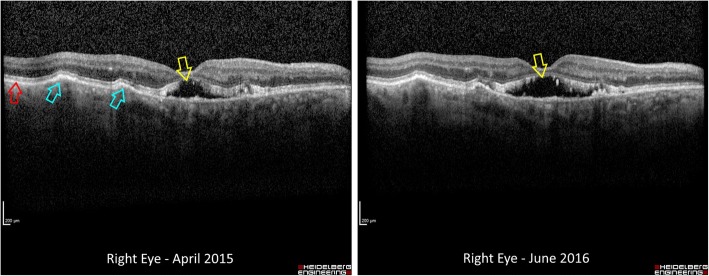
OCT of the Right eye demonstrating no response to intravitreal anti-VEGF therapy. (Yellow arrows mark sub-foveal space, Blue arrows mark drusen, Red arrow marks RPE)

In 2015 the OCT demonstrated an increasing sub-foveal space, raising a suspicion of CNVM. A loading phase of intravitreal anti vascular endothelial growth factor (anti-VEGF) was initiated in the form of Ranibizumab 0.5 mg. As there was no response to six Ranibizumab injections, treatment was subsequently switched to Aflibercept 2 mg with three further monthly doses. Again there was no improvement and treatment was ceased (Fig. 4).

Due to an unusual appearance and history when considering age related macular degeneration, a systematic review of his previous notes and images was performed as part of a retinal multi-disciplinary team.

**Fig. 5:**
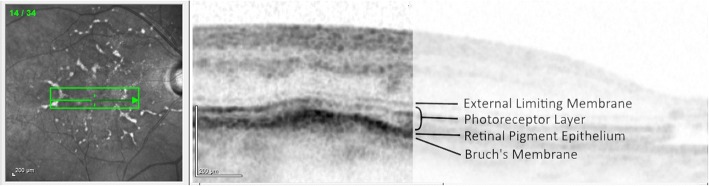
OCT cut through drusen showing preservation of the RPE and photoreceptors, with thickening of Bruch’s membrane

At the most recent visit (October 2016) the patient’s BCVA was recorded in the right eye as 49 ETDRS letters and awareness of hand movements only in the left eye. The fundus examination demonstrated multiple subretinal drusenoid deposits, which were autofluorescent, mainly at the posterior pole. Careful review of the OCT images demonstrated that the deposits were in Bruch’s membrane, with an intact Retinal Pigment Epithelium (RPE) (Fig. 5).

**Fig. 6:**
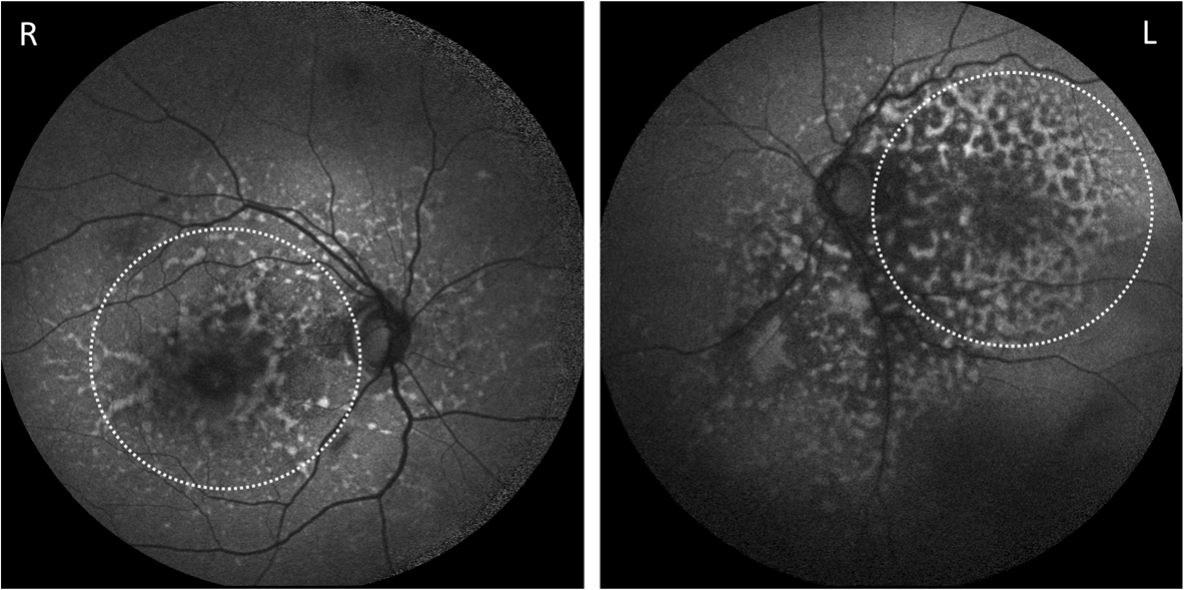
Bilateral Blue Auto-fluorescence (BAF) photographs demonstrating increased and decreased autofluorescence at the maculae (outlined by dotted white ellipses)

Autofluorescence imaging demonstrated large areas of increased and decreased autofluorescence involving the macula and spreading inferiorly (Fig. 6). OCT continued to demonstrate subfoveal hyporeflective areas. Pale optic discs were noted bilaterally, consistent with the long standing diagnosis of advanced glaucoma.

**Fig. 7:**
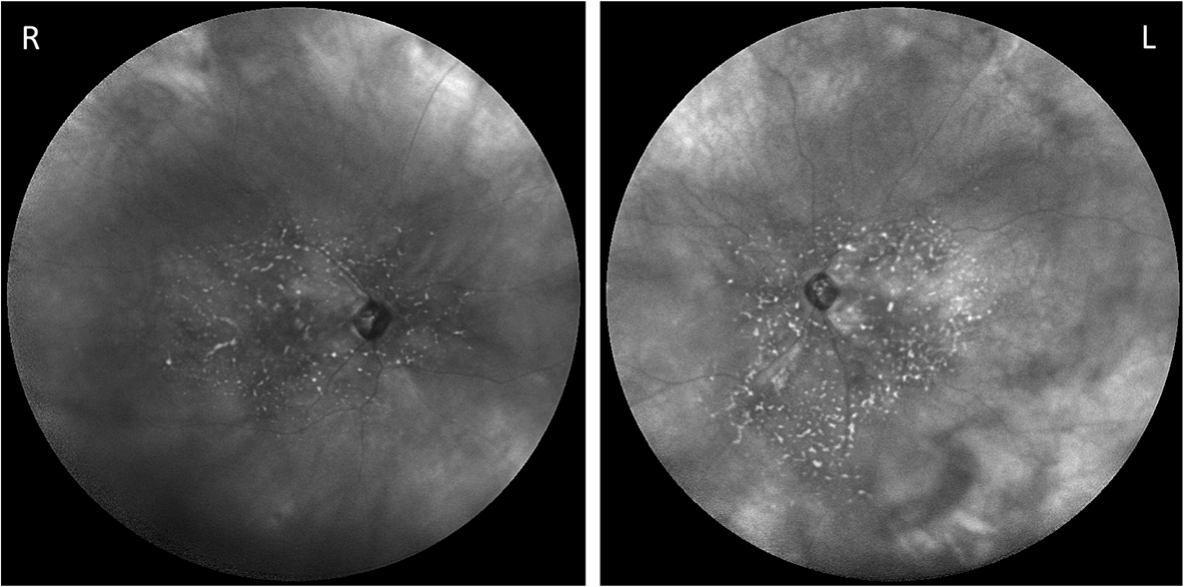
Bilateral wide angle infra-red fundus photographs demonstrating the limitation of the retinal changes to the posterior pole

Wide angle (102°) infra-red imaging demonstrates the limitation of the retinal disease to the posterior pole, involving the macula, crossing the vascular arcades and including the nasal peri-papillary region (Fig. 7).

**Fig. 8:**
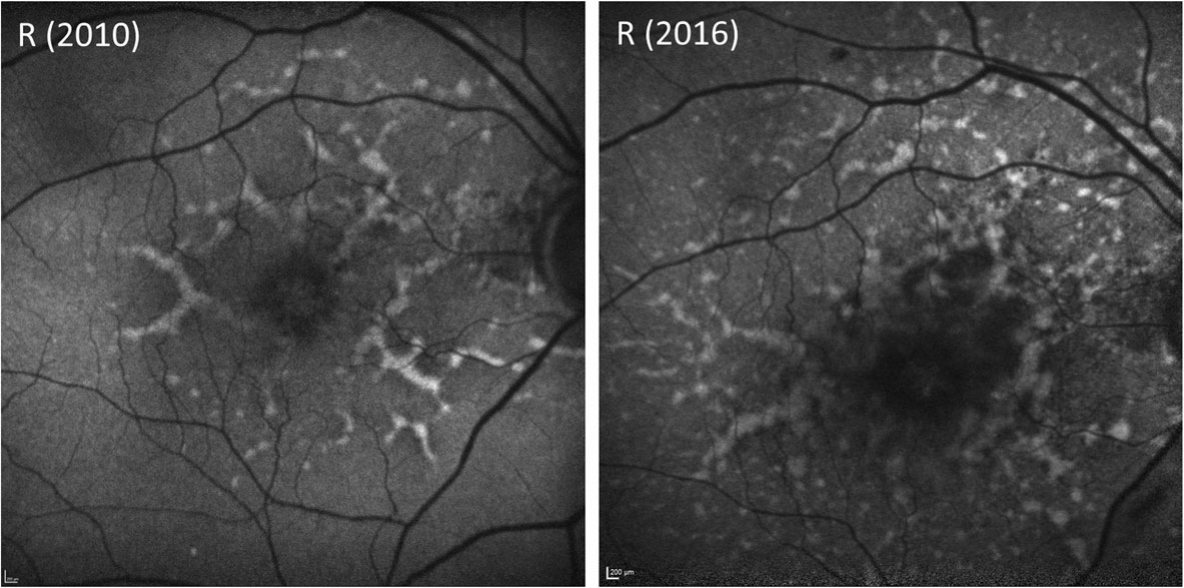
Comparison between the BAF appearance in 2010 and 2016 demonstrating an increasing number of sub-retinal deposits

The young age of presentation with drusen, recurrent kidney failure and increasing subretinal deposits (Fig. 8), led to the suspicion of this being a probable case of dense deposit disease. He is currently being tested for serum C3 levels and C3 nephritic factor as well as genetic testing for a variety of mutations in complement associated genes, with the results awaited.

## Discussion

Dense deposit disease (DDD) is a rare condition characterised by deposition of linear and focal electron-dense material in Bruch’s membrane and the lamina densa of the glomerular basement membrane [2, 3]. It is also referred to as membranoproliferative glomerulonephritis (MPGN) type II or mesangiocapillary glomerulonephritis type II with the retinal changes first being described by Duvall-Young, MacDonald and McKechnie in 1989 [3]. DDD is a subset of the C3 Glomerulopathies which have a prevalence estimated at 2–3 per 1,000,000 people [4].

The disease is usually diagnosed in children between the ages of 5 and 15 years old who present with haematuria, proteinuria, nephritic or nephrotic syndromes [5]. However, a retrospective cohort review in 2009 found that 39% of patients were not diagnosed until they were over 60 years of age [6]. The phenotype is non-specific, and includes the aforementioned renal problems, hypertension, low serum complement levels, raised C3 nephritic factor (C3NeF), retinal drusen, lipodystrophy, type 1 diabetes mellitus and monoclonal gammopathy of unknown significance [5]. Penetrance of all aspects of the phenotype is variable [7]. Although retinal drusen are present on examination in almost all cases of DDD, Savige et al. have noted that, as with our case, visual acuity is usually near normal even in the presence of abundant drusen [8]. The condition causes end stage renal disease in 50% of subjects within 10 years [1], and occasionally also impairs visual acuity and the field of vision [2, 8], predominantly by the development of subretinal neovascular membranes, macular detachment and central serous retinopathy [5]. Interestingly, Savige et al. noted that the six patients in their review all recorded nyctalopia [8] whilst our patient did not note reduced night vision. The findings of glaucoma and cataract in our patient, along with the complicated cataract surgery, are thought to be unrelated to his underlying diagnosis of DDD. The diagnosis of DDD can only be confirmed through renal biopsy, where deposits rich in C3 are found in the glomerular basement membrane [4].

The aetiology of DDD is unknown, although there is evidence that it is linked to the complement cascade and an increased activity of the alternative pathway, in particular, of C3 convertase. The most probable explanation for this is the presence of C3NeF, an autoantibody against C3 convertase [1]. Boon et al. have also identified that patients with DDD usually have a mutation of the complement factor H gene which renders it less effective in regulating the complement cascade [9]. This leads to the serum depletion of C3 and high levels of C3 within the deposits [2, 10]. This is in contrast to MPGN types I and III which have been found to have high levels of immunoglobulin G within the deposit structures.

The mutation of the complement factor H gene is thought to be the underlying cause of the retinal and glomerular basement membrane deposits. Both the RPE and the renal podocytes produce factor H, and therefore mutations in the gene cause deregulation of complement activation at the glomerular basement membrane and the Bruch’s membrane [11]. Damage to the Bruch’s membrane can then induce the growth of CNVMs, most probably through oxidative stress and inflammation, although the exact mechanisms are poorly understood [12]. The development of macular retinal detachment and central serous retinopathy are well documented in end stage renal disease, although the pathogenesis is poorly understood and may be related to an increase in choriocapillary permeability in the uraemic state [13].

There is currently no known treatment for DDD although multiple therapeutic regimes have been tried. Corticosteroids and immunosuppressants have been demonstrated to have no significant effect, and do not effectively suppress C3 transcription [5]. Renal transplant is often inevitable, but renal failure recurs in approximately 50% of patients [5]. Recent studies suggest that mycophenolate mofetil (MMF) can reduce the rate of progression to renal failure in C3 glomerulopathy by inhibiting inosine monophosphate dehydrogenase, however the benefit has not been demonstrated in DDD [4]. A trial of MMF is being considered for use with our patient to try to halt his progressive visual loss. An alternative therapy which has been shown to be effective in some patients is the anti-complement drug Eculizumab [7]. This agent targets the terminal part of the complement cascade, specifically inhibiting C5, although the clinical challenge will be identifying the cases which are appropriate for treatment [8].

This case report highlights the importance of considering the differential diagnosis and appropriate investigations in patients under 50 years of age who present with ocular drusen. Conditions which should be considered in such patients are listed in Table 1. The presentation of such a patient should warrant investigations so as to ensure that systemic causes have been excluded. This should include a urine dipstick and, if positive for protein or haematuria, an early referral to the renal team.

**Table 1:** Differential diagnoses of ocular Drusen in patients under 50 years of age

• Dense Deposit Disease (MPGN Type II or Mesangiocapillary Glomerulonephritis Type II)	
• Dominant Familial Drusen (Malattia Leventinese or Doyne’s Honeycomb Retinal Dystrophy)	
• Pattern Dystrophy	
• Bestrophinopathy	
• Sorsby Macular Dystrophy	
• Zermatt Macular Dystrophy	

Contrasting these differential diagnoses with the presentation in our patient identifies some key features which can be observed when reaching a final diagnosis. Dominant Familial Drusen would classically demonstrate a more regular, honeycomb, appearance to the soft drusen in contrast to our patient’s more diffuse and linear deposits. More significantly, IVFA demonstrates hyperfluorescence in familial dominant drusen, whilst our patient demonstrated a blocking defect and hypofluorescence. Pattern dystrophies are caused by lipofuscin accumulation in the RPE with subsequent loss of the photoreceptor cell layer [14]. The OCT in our case demonstrates preservation of all the retinal layers, with no thickening or deposition within the RPE layer. Bestrophinopathy has been found to present with central visual loss and night blindness. Autosomal dominant disease demonstrates classical vitelliform lesions, whilst the autosomal recessive type has neither drusen nor vitelliform lesions [15]. Finally Sorsby Macular Dystrophy would routinely present with bilateral CNVM and pseudo-inflammatory changes, whilst Zermatt Macular dystrophy affects the photoreceptors and demonstrates brightly hyperfluorescent lesions on IVFA in association with RPE atrophy [16, 17], none of which were seen in our patient.

The case presented here is of particular interest since it documents the course of the retinal changes associated with DDD over 30 years. Current literature has reviewed the original cohort of patients from Duvall-Young et al.’s paper over 10 years [1], and Jansen et al. reviewed a patient known to have DDD for 48 years but they only had access to 6 years of ophthalmological input [18]. Our case demonstrates the progressive nature of the retinopathy, and the late impact that this can have on visual function.

As no treatment exists for many of the causes of ocular drusen in young patients, adequate counselling should be offered to new patients, and regular follow up should be arranged to monitor any progression or change in the disease phenotype. Appropriate investigations including genetic testing and renal biopsy should be considered to confirm the diagnosis. Secondary disease, for example choroidal neovascular membranes, should be identified early and treated in line with local protocol.

## Conclusions

This case report documents the progression of ocular DDD over the course of 30 years, longer than any previous report. It highlights the importance of considering the systemic causes of ocular disease and ensuring appropriate investigations are conducted in a multi-disciplinary approach. All patients under 50 years of age who are noted to have retinal drusen should be investigated for renal disease at the time of presentation, and the drusen must not be attributed to age related macular degeneration. These patients should also be monitored regularly for the development of serous retinal detachment and choroidal neovascular membranes.

## Abbreviations

BAF: Blue Auto-fluorescence
BCVA: Best corrected visual acuity
C3NeF: C3 nephritic factor
CNVM: Choroidal neovascular membrane
DDD: Dense deposit disease
ETDRS: Early Treatment Diabetic Retinopathy Study
IVFA: Intravenous Fundus Fluorescein Angiogram
MMF: Mycophenolate mofetil
MPGN: Membranoproliferative glomerulonephritis
OCT: Optical Coherence Tomography
RPE: Retinal Pigment Epithelium
VEGF: Vascular endothelial growth factor
